# Mental Health Status of Patients Recovered from COVID-19 in Macau: A Cross-Sectional Survey

**DOI:** 10.3390/healthcare12212188

**Published:** 2024-11-04

**Authors:** Ting-Fai Man, Jing-Yu Zhu, Xi-Nan Song, Ying Bian

**Affiliations:** 1 Institute of Chinese Medical Sciences, University of Macau, Macau 999078, China; 2 State Key Laboratory of Quality Research in Chinese Medicine, University of Macau, Macau 999078, China; 3 Department of Public Health and Medicinal Administration, Faculty of Health Sciences, University of Macau, Macau 999078, China; 4 Faculty of Health Sciences and Sports, Macau Polytechnic University, Macau 999078, China

**Keywords:** mental health, Macau, sequelae, COVID-19, interventions, mental health crises

## Abstract

**Background/Objectives:** The COVID-19 pandemic has led to a global health crisis, impacting physical, and mental well-being, particularly among those who have recovered from the illness. This study aimed to assess the mental health status of patients recovered from COVID-19 in Macau, focusing on the impact of sequelae of COVID-19, and identifying demographic factors associated with poor mental health. **Methods:** A cross-sectional online survey was conducted involving 494 adults who had recovered from COVID-19, with 426 participants included in the final analysis. Mental health was evaluated using the 12-item General Health Questionnaire (GHQ-12), with scores ≥3 indicating poor mental health. **Results:** This study revealed a high prevalence of poor mental health, affecting 71.8% of the respondents. Binary logistic regression identified experiencing COVID-19 sequelae (OR = 5.727, 95% CI: 2.973–11.031), being in the age groups of 26–45 (OR = 4.227, 95% CI: 1.754–10.185), or 61, and above (OR = 18.072, 95% CI: 3.590–90.962), being male (OR = 0.501, 95% CI: 0.257–0.979), being married (OR = 5.714, 95% CI: 1.919–17.016), and dissatisfaction with family relationships (OR = 2.957, 95% CI: 1.228–7.119) as significant risk factors for poor mental health. **Conclusions:** This study underscores the critical need for targeted psychological support for patients recovered from COVID-19 in Macau, particularly for those with sequelae, and those in vulnerable demographic groups. The findings suggest that specific age groups and individuals with sequelae may face higher mental health risks, indicating the necessity for prioritized interventions.

## 1. Introduction

The outbreak of Coronavirus disease 2019 (COVID-19) has led to a global health crisis, with significant impacts on public health, economies, and societies worldwide [1,2,3,4,5,6,7,8,9,10]. Since its emergence in late 2019, efforts have been directed towards understanding the pathogenesis, clinical manifestations, and management of acute COVID-19. However, as the pandemic subsides, attention has turned towards investigating the long-term health implications for individuals who have recovered from the acute phase of the illness [3,11,12].

Research into post-COVID-19 complications, commonly referred to as long COVID or post-acute sequelae of SARS-CoV-2 infection (PASC), has gained momentum globally. Studies from various regions have reported a spectrum of persistent symptoms and health issues experienced by individuals post-recovery from COVID-19, highlighting the need for comprehensive understanding and targeted interventions. For instance, a study by Montani et al. revealed a high burden of multiorgan impairment in individuals recovering from COVID-19, emphasizing the diverse and complex nature of post-COVID-19 complications [13]. In China, the long-term effects of COVID-19 have manifested across multiple organ systems, including the respiratory, cardiovascular, neurological, and gastrointestinal systems. Patients recovering from COVID-19 frequently report persistent symptoms such as fatigue, muscle weakness, sleep disturbances, and cognitive impairments, which can last for months or even years [14]. This prolonged illness not only diminishes the quality of life for patients but also places a significant burden on the healthcare system and society. The rising healthcare costs and the need for long-term medical care and rehabilitation services underscore the necessity for ongoing research and effective management strategies to address the challenges posed by long COVID [15].

Furthermore, investigations into the underlying mechanisms and risk factors associated with post-COVID-19 complications have been documented in the literature. The study by Sudre et al. demonstrated the heterogeneity and multi-system involvement in long COVID, emphasizing the importance of multidisciplinary approaches in addressing these sequelae [16].

Globally, patients with long COVID face significant mental health challenges. A systematic review and meta-analysis by Seighali et al. found that approximately 23% of individuals with long COVID experience depression, 23% suffer from anxiety, and 45% have sleep disorders [17]. Qualitative research by Hossain et al. highlights the complex physical and psychosocial issues these patients endure, including persistent health problems, psychosocial crises, slow recovery, and changes in social support, emphasizing the need for comprehensive, multilevel interventions [18]. Additionally, a study by Wang et al. reported that one-third of patients with COVID-19 were diagnosed with neurological or psychological symptoms, such as anxiety, depression, and PTSD, within six months of contracting the virus [19].

In China, similar trends are observed. A study by Li et al. investigated the mental health of discharged patients with COVID-19 and found that many suffered from insomnia, depression, and anxiety [20]. These symptoms were particularly prevalent among women, the middle-aged and elderly, and those with underlying conditions. However, these symptoms significantly alleviated with rehabilitation and the transition from centralized quarantine to home isolation. Additionally, a two-year longitudinal study, conducted by Li Wang’s team at the Institutional Repository of Key Laboratory of Mental Health, investigated the long-term post-traumatic stress symptoms (PTSS) and their risk factors among COVID-19 survivors [21]. The study found that female survivors exhibited higher levels of PTSS and depression over two years, while male survivors showed higher levels of PTSS, depression, and anxiety at 2 months. COVID-19-related trauma and low social support were identified as risk factors for PTSS and negative emotions. Neuroimaging studies revealed increased amygdala activity in male survivors, which was associated with depressive symptoms and social support.

Understanding the mental health impacts on older adults across different cultures is crucial, especially in the context of the COVID-19 pandemic, which has exacerbated existing vulnerabilities and introduced new challenges. Older adults are particularly susceptible due to factors such as isolation, fear of the virus, and disruption of daily routines. Ahmed et al. (2024) examined the neurological and psychological impacts of COVID-19 on older adults in Bangladesh, revealing significant cognitive decline, such as memory loss and reduced attention span, along with heightened anxiety and depression symptoms post-COVID-19 infection [22]. Similarly, Giannouli and Giannoulis (2024) explored the use of technology to support older adults’ mental health in various cultural contexts, emphasizing the effectiveness of digital interventions like mental health apps, virtual reality therapy, and online counseling in reducing isolation and psychological distress [23]. Furthermore, Holland et al. (2024) focused on the psychological resilience and coping mechanisms of older adults during the pandemic [24]. They identified strong social support networks, positive attitudes, and flexible coping strategies as effective measures to support mental health. These studies underscore the importance of combining technological solutions and community support to effectively address the mental health needs of older adults in various cultural settings.

To date, there have been several domestic and foreign studies on the sequelae of COVID-19, yet there has been a lack of related research in Macau, a high-income region with high population density and a serious aging population. This paper aims to explore the relationship between the sequelae of COVID-19 and mental health status among people in Macau, providing empirical support for the need for supplementary psychological interventions to prevent the onset of mental health crises, especially among the most vulnerable groups.

## 2. Materials and Methods

### 2.1. Study Population

The research applies the Chinese online questionnaire platform form “Wen Juan Xing” to carry out the survey, informing people of survey-related matters and informed consent on the first page of the questionnaire; the members of research groups distribute the questionnaire quick response code(QR code) to the residents through the community social media such as WeChat groups, QQ groups, and WhatsApp; and participants scan the QR code to complete the questionnaire; they are asked to make a confirmation of the Survey Informed Consent Form before answering the questionnaire; all questions on the questionnaire are compulsory and need to be answered in full before submission. After the survey, the surveyors downloaded the survey data from “Wen Juan Xing” and conducted data collation and analysis. Responses to the questionnaire can be found in the Supplementary Materials.

Inclusion Criteria: (1) Individuals who have confirmed COVID-19 and have fully recovered; (2) aged over 18 years; (3) conscious, with normal reading and writing abilities, and capable of understanding the meaning of the questionnaire; (4) willing to voluntarily participate in this study after providing informed consent.

Exclusion Criteria: (1) Individuals with intellectual disabilities or cognitive impairments cannot participate in the survey; (2) individuals who are not willing to participate in the questionnaire survey will also be excluded.

The sample size was calculated using a single population proportion formula. We have taken the estimated proportion of poor mental health among patients recovered from COVID-19 as 30%, with a 95% confidence level, and a 5% margin of error.

$$n=\frac{{\left(z_{\alpha /2}\right)}^{2}.P\left(1-p\right)}{d^{2}}$$

The sample size was calculated as follows: n = (1.96)^2^ × 0.3(1 − 0.3)/(0.05)^2^ = 323. Finally, with the addition of a 15% contingency for non-response, the final sample size was 380 participants.

### 2.2. Measures

In this study, basic socio-demographic data were collected, including age, sex, education level, living status, and economic status. A structured questionnaire was also used to assess the mental health of participants. This questionnaire included specific questions regarding participants’ experiences with COVID-19. To evaluate the presence of sequelae of COVID-19, participants were asked, “Have you ever been infected with COVID-19 and recovered without experiencing any symptoms?” If participants answered “yes,” they were classified as having no sequelae of COVID-19. Conversely, if they answered “no,” they were classified as having sequelae of COVID-19. 

Mental health in the past week was evaluated with validated Chinese versions of the General Health Questionnaire 12-item version (GHQ-12) [25,26,27]. This questionnaire consists of 12 items and is scored according to the World Health Organization’s bimodal scoring method, whereby “less than usual” and “no more than usual” were both worth 0 points, and “rather more than usual” and “much more than usual” were each worth 1 point. The lowest of the 12 items scored 0 and the highest scored 12, with higher scores indicating poorer mental health. The cut-off score is 3, i.e., <3 indicates good mental health and ≥3 indicates poor mental health. The Cronbach’s alpha coefficient of the scale in this study was 0.746.

### 2.3. Sequelae

Individuals infected with COVID-19 may experience enduring post-infection sequelae, commonly referred to by various terms, including long COVID or long-haul COVID. A Delphi process, led by the World Health Organization (WHO), was assembled to reach a consensus definition for the condition in question [28]. Over two evaluation rounds, 14 domains, and 45 items were assessed, resulting in a final consensus definition for adults. The post-COVID-19 condition is defined by symptoms occurring in individuals with a history of probable or confirmed SARS-CoV-2 infection, typically emerging three months after the initial infection and lasting for at least two months without an alternative diagnosis. Common symptoms of this condition include, but are not limited to, fatigue, shortness of breath, and cognitive dysfunction, which generally have an impact on daily functioning. Considering the complexity of medical terms, the public may struggle to accurately determine whether they are experiencing post-COVID-19 sequelae. Therefore, in this study, sequelae are defined as symptoms reported by respondents that emerged following their recovery from COVID-19, including both physiological and psychological adverse effects, including insomnia, loss of appetite, and ageusia. If they self-reported the above conditions, they were defined as having sequelae; otherwise, they were defined as having no sequelae.

### 2.4. Covariates

The covariates in this study were categorized into demographic variables and risk factors. Demographic variables included (1) Age (18–25/26–45/46–60/61 or above), (2) Gender (male or female), (3) Marital Status (divorced/widowed/unmarried/married), and (4) Education (junior high school degree or below/high school degree/college degree/bachelor’s degree or above). Risk factors included income, which is categorized into five groups: 5000–10,000, 10,000–15,000, 15,000–20,000, 20,000–30,000, and above. The unit of income is Macau Patacas (MOP). Satisfaction is defined as whether one is satisfied with the relationships with family members, including parents, siblings, spouses, and children. Previous research indicates that good familial relationships positively influence both mental and physical health outcomes [29,30,31]. In this study, participants were categorized as ‘satisfied’ with their familial relationships if they reported being satisfied and ‘dissatisfied’ otherwise.

### 2.5. Statistical Methods

SPSS (version 23.0, IBM Corporation, Armonk, New York, USA) was used for data analysis, and descriptive statistics were used to analyze the situation of COVID-19 infection, sequela, and general mental health in the participants; χ^2^ was used to test and compare the differences in the rate of COVID-19 infection in different participants; correlation analysis was used to understand the relationship between COVID-19 sequela and general mental health.

Depending on the outcome variables, we used a binary logistic regression model to analyze the relationship between sequelae and mental health. Specifically, the statistical model is as follows:

(1) $$\mathrm{logit}(P)=\beta_{0}+\beta_{1}X_{1}+\beta_{2}X_{2}+...+\beta_{k}X_{k}$$

In the equation, *P* represents the probability of poor mental health. *β*_0_ is the intercept term of the model. *X*_1_ represents sequelae variables. *p* < 0.05 was considered statistically significant. To account for the influence of confounders on outcomes, we adjusted the model for demographic information as well as participants’ income and satisfaction. In this study, Model 1 adjusted for age, gender, and marital status to account for the influence of these demographic variables on the probability of poor mental health. Model 2 included all adjustments from Model 1 and additionally controlled for education, income, and satisfaction with family relationships to provide a more comprehensive adjustment. This approach allows for a more accurate assessment of the independent impact of COVID-19 sequelae on mental health by controlling for various confounding factors.

The odds ratio (OR) is a key measure used in logistic regression to quantify the strength of the association between a predictor variable and the outcome. An OR greater than 1 indicates an increased likelihood of the outcome occurring with the predictor, while an OR less than 1 indicates a decreased likelihood. In the context of our study, an OR greater than 1 suggests that the predictor variable, such as the presence of COVID-19 sequelae, is associated with a higher likelihood of poor mental health. Conversely, an OR less than 1 indicates that the predictor variable is associated with a lower likelihood of poor mental health. This measure helps us understand the impact of various factors on mental health outcomes and guides the development of targeted interventions.

## 3. Results

### 3.1. Demographic Characteristics of the Respondents

This study revealed a sample with a nearly balanced gender ratio, with a predominance of young people (47.4% were aged 18–25). Educational backgrounds were generally high, with more than half (52.3%) of the participants holding a bachelor’s degree or higher. Marital status showed a preference for single people (52.6% were unmarried), while the income distribution indicated a sizeable middle-income group (30.8% in the 10,000–15,000 mop range). Overall, the majority of participants (65.3%) were satisfied with their family life, but a significant number (34.7%) were still dissatisfied.

### 3.2. The Situation of COVID-19 Infections and Sequelae

Almost half of the people (58.7%) in the survey were infected with COVID-19 twice or more and 176 (41.3%) once only. About half reported having sequelae, while about half reported no sequelae.

### 3.3. Comparison of Poor Mental Health in Recovered Patients with Different Variables

The score of GHQ-12 of the respondents was 4.47 ± 2.78 of 426 recovered patients, with a minimum of 0.00 and a maximum of 11.0. Of the 426 respondents, 306 (71.8%) were classified as having poor mental health, meaning that their score was greater than or equal to 3.00.

The chi-square test results showed that there was no statistically significant difference in the prevalence of poor mental health among different income levels (*p* > 0.05); other variables including gender, age, education level, marital status, and sequelae, were statistically significant (all *p* < 0.01) (Table 1).

### 3.4. Analysis of the Correlation between Basic Socio-Demographic Variables, COVID-19 Sequelae, and Mental Health

**Figure 1:**
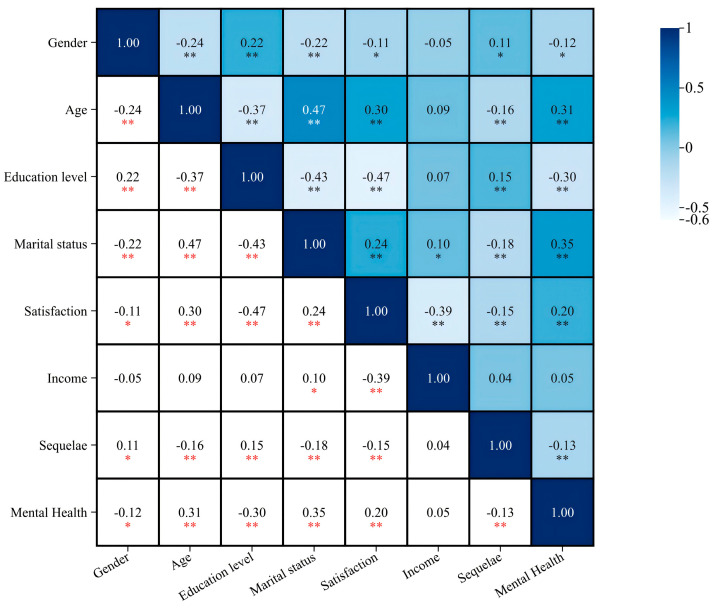
Analysis of the correlation between basic socio-demographic variables, COVID-19 sequelae, and mental health in the respondents. Note: * *p* < 0.05; ** *p* < 0.01.

The correlation analysis showed that apart from income, other variables show a correlation with mental health. Age, satisfaction, and marital status were significantly and positively correlated with mental health (all *p* < 0.01), r = 0.31, 0.20, 0.35; gender was significantly and negatively correlated with mental health (*p* < 0.05), r = −0.12, whereas sequelae were significantly and negatively correlated with mental health (*p* < 0.01), r = −0.13 (Figure 1).

### 3.5. Binary Logistic Regression Analysis of the Effect of Sequelae on Mental Health

In Table 2, the binary logistic regression analysis indicated that the presence of COVID-19 sequelae significantly increases the likelihood of poor mental health across all models. In the crude model, individuals with sequelae were found to be more than five-fold (OR = 5.339, 95% CI: 3.284–8.680) more likely to experience poor mental health. This association remained robust after controlling for confounding variables such as age, gender, and marital status in Model 1 (OR = 5.244, 95% CI: 2.857–9.628) and further adjustments for education, income, and satisfaction with family relationships in Model 2 (OR = 5.727, 95% CI: 2.973–11.031) (Table 2).

**Figure 2:**
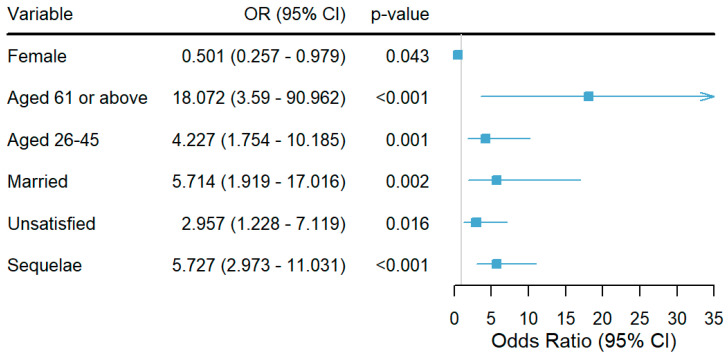
Forest plot showing binary logistic regression. Notes: Females (Ref: males), individuals aged 61 or above and 26–45 (both Ref: aged 18–25), married individuals (Ref: unmarried), unsatisfied individuals (Ref: satisfied), and those with sequelae (Ref: no sequelae) are presented. Values are shown as odds ratios (OR) with 95% confidence intervals (CI), and statistical significance is indicated by *p*-values, with *p* < 0.05 considered as the threshold for significance.

The results of the regression analysis showed that females are less likely than males to have poor mental health (OR = 0.501, 95% CI: 0.257–0.979), indicating that being female was a protective factor against poor mental health. Being aged 26–45 (OR = 4.227, 95% CI: 1.754–10.185) and 61 or above (OR = 18.072, 95% CI: 3.590–90.962) were risk factors for poor mental health. Being married was a risk factor for poor mental health (OR = 5.714, 95% CI: 1.919–17.016). Dissatisfaction with family relationships was a risk factor for poor mental health (OR = 2.957, 95% CI: 1.228–7.119). Having sequelae was a risk factor for poor mental health (OR = 5.727, 95% CI: 2.973–11.031) (Figure 2).

## 4. Discussion

In this study, the rate of poor mental health in respondents is 71.8%. It indicates that most of the respondents suffer from mental health. Of these, respondents with sequelae were generally more likely to have poor mental health. It is assumed that this is due to the negative impact of sequelae, both physical and psychological, such as forgetfulness, insomnia, and pain [32]. A study showed that the long COVID-19 cohort exhibited significantly higher anxiety scores compared to those who never acquired COVID-19 [33]. While we identified a significant association between COVID-19 sequelae and mental health status, it is important to note that other potential confounders may have influenced this result. These factors include, but are not limited to, participants’ socioeconomic status, level of social support, and lifestyle. Individuals with lower socioeconomic status may be at greater mental health risk [34]. In addition, lower levels of social support and unhealthy lifestyles (e.g., lack of exercise and unbalanced diet) may also exacerbate mental health problems [35,36].

This study’s results revealed significant differences in the prevalence of poor mental health among individuals of varying genders and ages. Males, the youth, and the elderly were found to have a higher likelihood of experiencing poor mental health. Therefore, it is recommended that more attention be given to these groups in the prevention and management of mental health crises. This study found that females were significantly less likely to report poor mental health compared to males. This result contradicts many studies that suggest females are more vulnerable to mental health issues during crises [37]. Possible explanations include the specific cultural context and gender-specific coping mechanisms. In Macau, females may be more inclined to seek social support and engage in emotional communication, which can help alleviate psychological stress. Additionally, the traditional division in Chinese families, where men are typically responsible for external affairs and women for domestic affairs, may play a role. According to the Social Welfare Bureau of the Macau Special Administrative Region’s ‘Macau Women’s Current Situation Report 2022’, the labor force participation rate of females was lower than that of males from 2017 to 2021. It suggests that females in Macau may be more likely to engage in domestic labor rather than seek employment outside the home. This division can provide women with a stable and supportive home environment, enabling them to exhibit greater resilience and adaptability in the face of crises. On the other hand, males may face greater stress due to the expectation of being the primary breadwinner. During the pandemic, the risk of unemployment and loss of income can significantly impact their mental health, as they may feel a heightened sense of responsibility and pressure to provide for their families [38,39]. Furthermore, it was found that women, especially older women, compared to older men, have higher self-estimations. According to a study by Giannouli et al., older women tend to have higher self-estimations of their cognitive abilities and emotional intelligence compared to older men. This higher self-esteem and positive self-perception can act as protective factors against poor mental health, making them less likely to experience mental health issues [40]. However, this hypothesis requires further research to verify.

In this study, people aged 61 or above have a higher probability of poor mental health compared to those aged 18–25. It is evident that the elderly are a vulnerable group to mental health crises. Long COVID is a particular concern for older individuals, aged 65 years or older, who are at a higher risk of experiencing persistent symptoms associated with COVID-19 compared to younger individuals [41]. A study investigating the quality of life and well-being during the COVID-19 pandemic, and the result showed that chronic health conditions and the COVID-19 pandemic had a negative impact on mental health and were associated with lower quality of life and well-being [42]. According to demographic statistics for the 4th quarter of 2022 in Macau, the proportion of the elderly population (aged 65 and above) was 13.3%, meaning that Macau is an aging society. It is suggested that the authorities should allocate more resources to avoid the emergence of mental health crises by providing a 24 h helpline or regular psychological counseling services [43]. These measures would enable the timely identification of at-risk individuals and their referral to medical institutions for assistance.

This study represents the first investigation conducted in Macau to investigate the characteristics of Long COVID and its impact on mental health. The information provided can be used by public health officials and policymakers as part of post-pandemic recovery plans. The limitations of this study are that the sample size is not large compared with other similar studies. According to the result of binary logistics regression, it can be seen that the 95% confidence interval of OR of people aged 61 or above is too wide, which may be due to a small sample size or high variability in the data. Additionally, since the sampling method in this study is convenience sampling, it may lead to biased results because it relies on selecting individuals who are easily accessible or readily available rather than using a random or representative sampling method.

The recruitment strategy in this study, which relied on convenience sampling and online questionnaires distributed via social media, may limit the generalizability of the findings. Convenience sampling can introduce selection bias, as it may not accurately represent the broader population of patients recovered from COVID-19 in Macau. Online questionnaires may exclude individuals without internet access or who do not use social media, skewing the sample towards a younger, more tech-savvy demographic [44]. Additionally, Macau’s unique geographical and demographic characteristics, such as its high population density and significant aging population, further limit the applicability of the results to other regions. These factors suggest that the findings may not be generalizable to different populations. Future research should employ random sampling and mixed-methods approaches, including telephone interviews or in-person surveys, to improve the representativeness and applicability of the findings [45].

## 5. Conclusions

This study investigated the mental health status of individuals in Macau who have recovered from COVID-19, focusing on the impact of sequelae. The findings indicate that a significant proportion of recovered patients experience poor mental health, with sequelae being a major contributing factor.

This study also identified that age, gender, marital status, and satisfaction with family relationships are significant factors influencing mental health. Older adults (61 years and above) and those aged 26–45 were at higher risk of poor mental health. Additionally, males and individuals dissatisfied with their family relationships were more likely to experience mental health issues.

These results highlight the need for targeted psychological interventions for COVID-19 survivors, particularly those with sequelae and those in vulnerable demographic groups. Future research should focus on understanding the mechanisms behind these associations and developing effective intervention strategies.

## Figures and Tables

**Table 1:** Comparison of poor mental health in recovered patients with different variables.

Variables, *n* (%)	Total Sample	Poor Mental Health	*χ* ^2^	*p*-Value
	(*n* = 426)	(*n* = 306)		
Gender			37.118	<0.001 ***
Female	212 (49.8%)	124 (40.5%)		
Male	214 (50.2%)	182 (59.5%)		
Age			109.638	<0.001 ***
18–25	202 (47.4%)	97 (31.7%		
26–45	85 (20.0%)	75 (24.5%)		
46–60	48 (11.3%)	45 (14.7%)		
61 or above	91 (21.4%)	89 (29.1%)		
Education			90.947	<0.001 ***
Junior high school degree or below	18 (4.2%)	17 (5.6%)		
High school degree	60 (14.1%)	55 (18.0%)		
College degree	125 (29.3%)	118 (38.6%)		
Bachelor’s degree or above	223 (52.3%)	116 (37.9%)		
Marital status			112.29	<0.001 ***
Unmarried	224 (52.6%)	112 (36.6%)		
Married	153 (35.9%)	148 (48.4%)		
Divorced	37 (8.7%)	36 (11.8%)		
Widowed	12 (2.8%)	10 (3.3%)		
Satisfaction with family relationships			36.452	<0.001 ***
Satisfied	278 (65.3%)	173 (56.5%)		
Unsatisfied	148 (34.7%)	133 (43.5%)		
Income			14.072	0.003 **
5000–10,000 mop	70 (16.4%)	50 (16.3%)		
10,000–15,000 mop	131 (30.8%)	81 (26.5%)		
15,000–20,000 mop	106 (24.9%)	76 (24.8%)		
20,000–30,000 mop	119 (27.9%)	99 (32.4%)		
Sequelae				
Yes	213 (50.0%)	186 (60.8%)	50.535	<0.001 ***
No	213 (50.0%)	120 (39.2%)		

Notes: * *p* **<** 0.05; ** *p* **<** 0.01; *** *p* **<** 0.001.

**Table 2:** Binary logistic regression analysis of the effect of sequelae on mental health.

Variable	Crude Model		Model 1		Model 2	
	OR	95% CI	OR	95% CI	OR	95% CI
Sequelae						
No	Ref	-	Ref	-	Ref	-
Yes	5.339 ***	(3.284, 8.680)	5.244 ***	(2.857, 9.628)	5.727 ***	(2.973, 11.031)
Age						
18–25			Ref	-	Ref	-
26–45			4.254 **	(1.855, 9.756)	4.227 ***	(1.754, 10.185)
46–60			3.79	(0.901, 15.946)	2.667	(0.615, 11.566)
61 or above			29.388 ***	(6.340, 136.229)	18.072 ***	(3.590, 90.962)
Gender						
Female			Ref	-	Ref	-
Male			0.442 **	(0.240, 0.816)	0.501 *	(0.257, 0.979)
Marital status						
Unmarried			Ref	-	Ref	-
Married			13.492 ***	(4.906, 37.105)	5.714 **	(1.919, 17.016)
Divorced			17.529 **	(2.135, 143.944)	8.189	(0.971, 69.085)
Widowed			1.047	(0.166, 6.601)	0.315	(0.043, 2.327)
Education						
Junior high school degree or below					Ref	-
High school degree					0.954	(0.086, 10.531)
College degree					3.442	(0.296, 40.056)
Bachelor’s degree or above					0.678	(0.070, 6.608)
Satisfaction with family relationships						
Satisfied					Ref	-
Unsatisfied					2.957 *	(1.228, 7.119)
Income						
5000–10,000 mop					Ref	-
10,000–15,000 mop					0.678	(0.256, 1.798)
15,000–20,000 mop					0.776	(0.277, 2.171)
20,000–30,000 mop					2.025	(0.726, 5.644)

Crude model: No adjustments. Model 1: Partially adjusted model, adjusting for age, gender, and marital status. Model 2: Fully adjusted model, additionally adjusting for education, income, and satisfaction. OR: odds ratio; CI: confidence interval. * *p* < 0.05, ** *p* < 0.01, *** *p* < 0.001.

## Data Availability

The data supporting the findings of this study are available within its Supplementary Materials.

## Supplementary Materials

The following supporting information can be downloaded at: https://www.mdpi.com/article/10.3390/healthcare12212188/s1.
